# Combination of Mustardé cheek advancement flap and paramedian forehead flap as a functional and aesthetic alternative

**DOI:** 10.1093/jscr/rjad019

**Published:** 2023-01-31

**Authors:** Paola Solis-Pazmino, Richard Godoy, Eduardo Pilatuña, Carla Rocha, Cristhian García

**Affiliations:** Head and Neck Surgery, Instituto de la Tiroides y Enfermedades de Cabeza y Cuello (ITECC), Quito, Ecuador; Head and Neck Surgery, Instituto de la Tiroides y Enfermedades de Cabeza y Cuello (ITECC), Quito, Ecuador; Head and Neck Surgery, Instituto de la Tiroides y Enfermedades de Cabeza y Cuello (ITECC), Quito, Ecuador; Head and Neck Surgery, Instituto de la Tiroides y Enfermedades de Cabeza y Cuello (ITECC), Quito, Ecuador; Head and Neck Surgery, Instituto de la Tiroides y Enfermedades de Cabeza y Cuello (ITECC), Quito, Ecuador

## Abstract

Depending on the size and location, defects resulting from the surgical procedure due to basal cell carcinoma (BCC) may be challenging to reconstruct. A combination of more than one flap type might be necessary for moderate to large-sized defects, especially in face lesions. We present a patient with a large BCC in the nasal region, successfully closed using a combination of rotation and advancement flaps. The patient showed excellent functional and cosmetic outcomes.

## INTRODUCTION

Basal cell carcinoma (BCC) is a nonmelanocytic skin cancer arising from the epidermis's basal layer [1]. According to the National Comprehensive Cancer Network (NCCN), the goal of treatment for BCC is eradicating the tumor to avoid the locally invasive and destructive behavior, with maximal preservation of function and physical appearance [2]. In most advanced BCC cases, primary closure from the surgical procedure is complex and thus requires a skin flap or graft [3]. This report describes a patient with a significant defect on the nose following BCC excisional surgery, which was successfully closed using a combination of rotation and advancement flaps. The patient showed excellent functional and cosmetic outcomes.

## CASE PRESENTATION

An older woman presented to our center with a 5-year history of a persistent nasal vestibular lesion. It was treated with multiple cauterizations without a total improvement until then. There was no associated pain, bleeding, or drainage. She denied family history, risk factors, or systemic diseases associated with this skin pathology.

**Figure 1:**
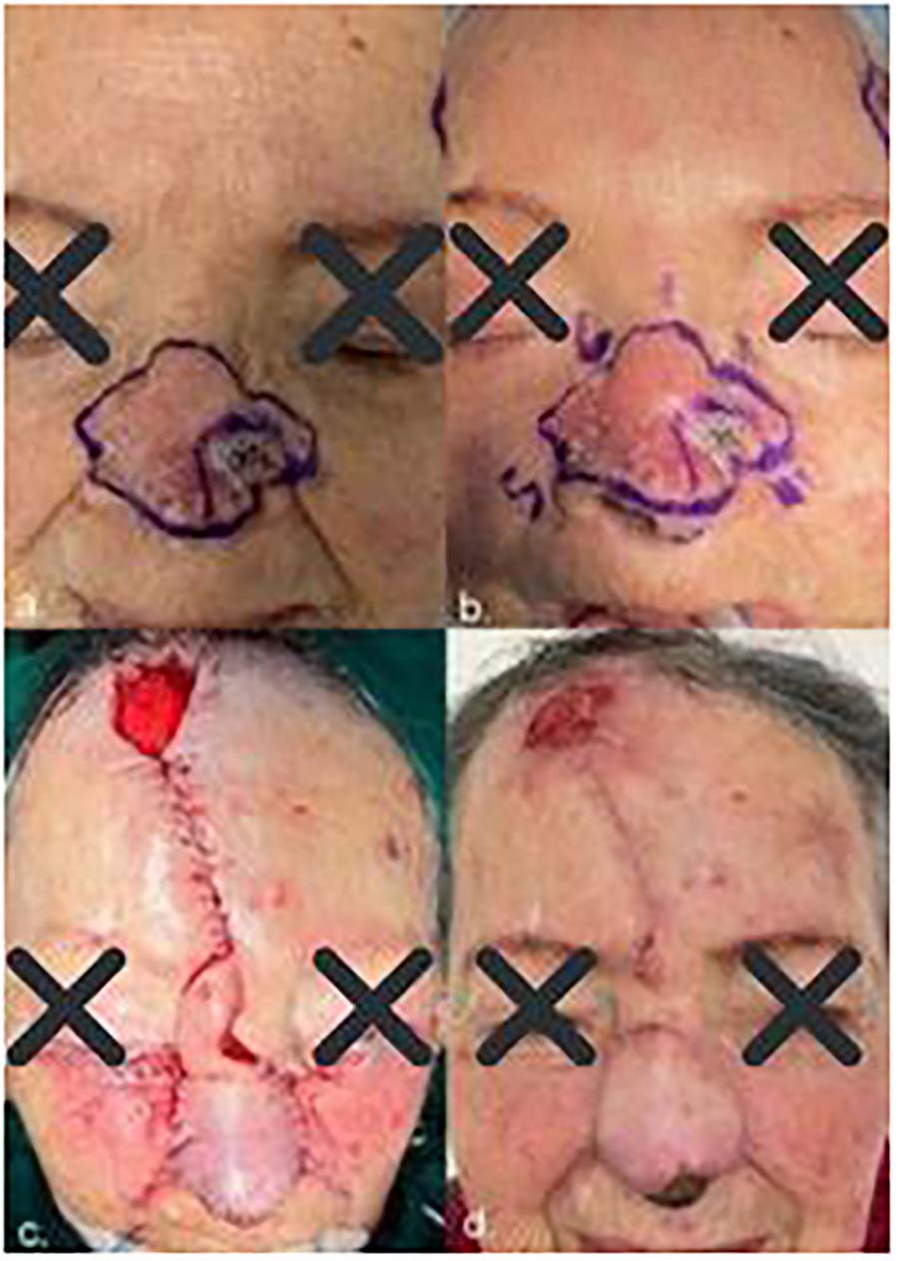
(**a**) Initial lesion; (**b**) clockwise order lesion; (**c**) immediate post-operative result and (**d**) follow-up after 4 weeks showed excellent functional and cosmetic results.

Physical examination revealed a 4 cm × 3 cm desquamated plaque with multiple pustules and a small deep ulcered area on the left nasal vestibule and overlying telangiectasia (Fig. 1a).

### Investigations if relevant

A skin biopsy confirmed the clinical suspicion that this patient had nodular BCC, also known as rodent ulcer.

### Treatment

**Figure 2:**
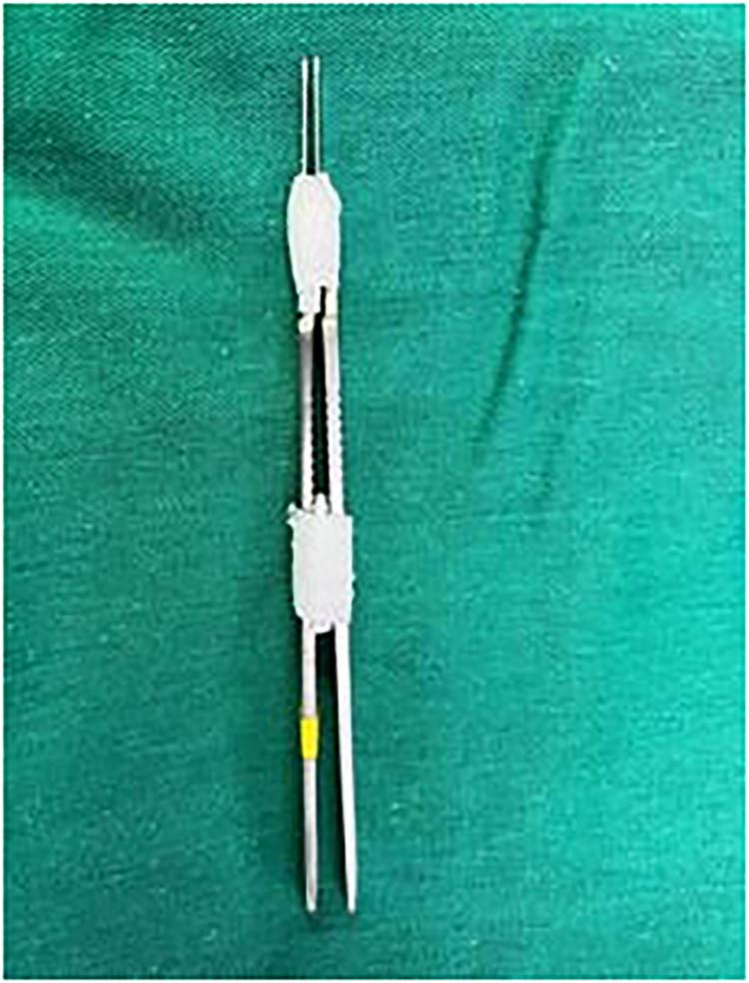
Double scalpel blade.

The patient underwent surgery under general anesthesia. During the surgery, we sent an intraoperative frozen section (IFS), 6 margins were taken in 10-mm strips each (Fig. 1b) and two of the 7 tissue samples were reported positive. Made do with a double scalpel blade (Fig. 2) numbered by segments following clockwise order.

Meanwhile, the defect reconstruction was done after histopathological examination confirmed tumor-free margins. A local skin flap, a paramedian forehead flap, transferred full-thickness skin and subcutaneous tissue into the surgical defect and maintained the local blood supply via a vascular pedicle connected to the donor site. Then, the bilateral advancement flap (Mustardé) was slid into the defect where the incisions were made tangentially to the defect to free neighboring tissue, with the wound edge acting as the free margin of the flap. Finally, for the frontal opening graft tissue, we used a pressure dressing on cutaneous grafts using a gauze wad (Brown Curative) (Fig. 1c).

### Outcome and follow-up

After 6 weeks, the pedicle of the flap was detached, and the flap was inset in the final position (Fig. 1d). The patient responded well to the treatment, remains under close outpatient review and has no other symptoms following surgery.

## DIFFERENTIAL DIAGNOSIS

**Figure 3:**
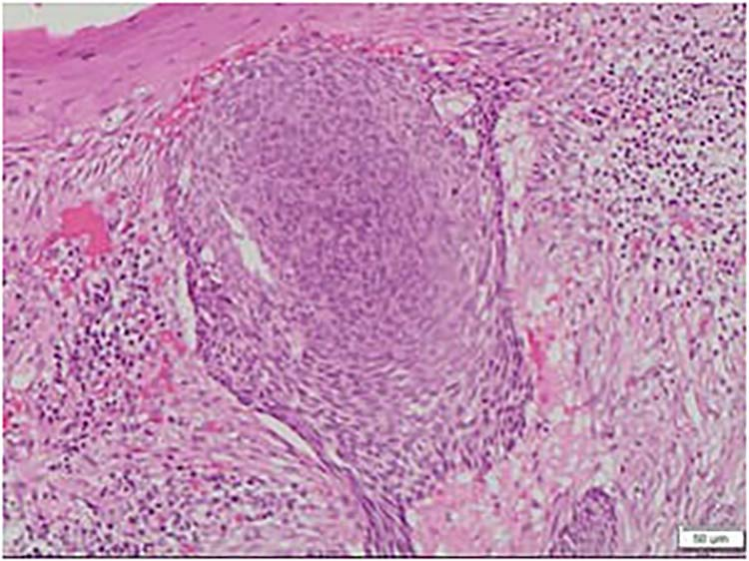
Microscopic view of the BCC, nodular type: note the large nest of basaloid cells centered in the dermis with cleft retraction, peripheral palisading, mitotic activity and cytological atypia.

Some histopathological differential diagnoses include basaloid tumors. Trichoepithelioma is a benign adnexal tumor, which may show morphologic overlap and unchanging anatomical localization as BCC. However, the prominent cleft retraction, peripheral palisading, significant mitotic activity, cytological atypia (Fig. 3) and necrosis seen in this case are incompatible with Trichoepithelioma. On the other hand, basaloid squamous cell carcinoma is characterized by paradoxical maturation and areas with convenient features such as keratinization and keratin pearls. These are not seen in our case.

## DISCUSSION

BCC is the most common skin cancer worldwide. According to the American Cancer Society, about 5.4 million basal and squamous cell skin cancers are diagnosed yearly in the US, with BCC constituting most cases: 8 out of 10 [4]. BCC's higher incidence rates are in countries closer to the equator and near the hole in the ozone layer above the Antarctic [5]. Quito, the capital of Ecuador, is just 15.86 mi (25.52 km) south of the equator [6] and reported the highest number of cases [7].

According to the NCCN, the goal of treatment for BCC is eradicating the tumor using surgery with maximal preservation of function and physical appearance [2]. It allows excision margin control and shows a low risk of recurrence. This approach varies according to tumor size, depth, and location. It allows the microscopic examination of the entire surgical margins during surgery so that the extent of excision can be defined precisely [8]. In our case, we used a 10 mm strip margin with a double scalpel blade to ensure the quality of the biopsy sample and, subsequently, the oncological results. The oncological accepted margins depend on the risk group and the tumor size. For primary well-demarcated BCCs smaller than 2 cm, in the low-risk group, 3 mm gives satisfactory results. Whereas, in the high-risk group, and for lesions larger than 2 cm, a 4–6 mm margin is suggested for getting clear margins [9].

The face is the site most affected by BCC, and after the surgical resection, an ideal facial reconstruction must provide functionality and an appropriate cosmetic appearance. Combining a local and an advancement flap can be further done to achieve this goal [9]. Reconstruction of our patient used a combination of the paramedian forehead flap, developed by Sushruta Samita in ancient India. The second flap was a cheek rotational (Mustardé) flap designed in the 1960s. Some studies [8, 10] mentioned that the combination of both flaps has a significant benefit in closing large defects in terms of mobility of mid and lateral cheek due to the unique features of the skin, excellent color and texture match, wide-based pedicle flap with appropriate vascularity and good aesthetic outcome. A large study of 116 patients demonstrated a low rate of recurrence of BCC in those cases where flaps and grafts were used in the surgical reconstruction with pre-established margins by the conventional method [11].

### Learning points/take home messages

- BCC is the most common skin malignancy presented on sun-exposed areas such as the face.
- Oncological accepted margins depend on the risk group and the tumor size. For primary well-demarcated BCCs smaller than 2 cm, in the low-risk group, a margin of 3 mm gives satisfactory results.
- Combining skin flap techniques such as the paramedian forehead flap and the cheek rotational (Mustardé) flap showed excellent functional and cosmetic outcomes.

## CONFLICT OF INTEREST STATEMENT

None declared.

## FUNDING

None.
